# Identifying gaps between perceived and actual intakes in Finnish adults: self-assessment of macronutrient intakes in relation to nutrition recommendations is challenging

**DOI:** 10.29219/fnr.v69.10535

**Published:** 2025-02-04

**Authors:** Sari Bäck, Tiina Pellinen, Essi Päivärinta, Petra Rautio, Antti Isokangas, Maijaliisa Erkkola, Anne-Maria Pajari

**Affiliations:** 1 University of Helsinki, Department of Food and Nutrition, Helsinki, Finland;; 2 Makery Oy, Helsinki, Finland

**Keywords:** actual dietary intake, food-related behaviour, nutrition knowledge, nutrition literacy, perceived dietary intake

## Abstract

**Background:**

Population adherence to nutrition recommendations measured by dietary surveys is well known, but people’s perceptions in adherence to nutrition recommendations are less explored. For macronutrients, nutrition recommendations suggest broad intake ranges.

**Objective:**

To study individuals’ perceptions of their macronutrient intakes compared to the Nordic Nutrition Recommendations and discrepancies between perceived and actual macronutrient intakes.

**Design:**

The ScenoProt trial investigated nutritional and health effects of replacing animal-source proteins with plant-source proteins in Finnish adults (*n* = 102, 78% women, mean age 47 years). This cross-sectional sub-study utilized data collected at the baseline of the trial. Participants’ perceptions of intakes and sources of carbohydrates, fibers, fats, and proteins were collected by a questionnaire developed for the study. Actual macronutrient intakes and sources were assessed with 4-day food records. Logistic regression analysis was used to examine associations between sociodemographic factors and the capacity to self-assess macronutrient intakes relative to the nutrition recommendations.

**Results:**

Discrepancies were found in relative proportions of three categories, below/according to/above the recommendation, between perceived and actual macronutrient intakes regarding the nutrition recommendations. Participants rated themselves rather according to or above than below the recommendation. The most distinct gap was discovered between perceived and actual carbohydrate intakes, for example, proportions of participants being below the recommendation: 8% measured as perceived intake; 70% measured as actual mean intake. Gaps were also observed for fat and protein. Cereals were one of the most common protein sources but only 8% of the participants named them. No associations emerged between age, gender, or education (46% with a master’s degree or higher) and the capacity to self-assess macronutrient intakes.

**Discussion & conclusions:**

Our study suggests that self-assessment of macronutrient intakes is challenging. Misperceptions can be obstacles in shifting to healthier and more sustainable diets. Interpretation of nutrition recommendations to the public could still be improved.

## Popular scientific summary

- Nutrition recommendations inform intake ranges for macronutrients. People’s perceptions in adherence to these recommendations are not well known.
- Perceptions of carbohydrate, fiber, fat, and protein intakes were compared to the intake ranges of the Nordic Nutrition Recommendations. Perceived macronutrient intakes were compared with actual measured intakes.
- Self-assessment of macronutrient intakes was challenging especially for carbohydrates. Cereals were not identified as a protein source. Misperceptions can hinder the transition to healthier and more sustainable diets.

Nutrition recommendations and food-based dietary guidelines (FBDGs) are evidence-based tools for food choices meeting nutritional needs while also promoting public health by lowering the risk of diet-related chronic diseases (1, 2). Nutrition recommendations provide nutrient-based guidance mainly for population-level purposes, for example, in policy-making, dietary planning, and the health sector (3). FBDGs, in turn, give information about foods and food groups with the indicative consumption amounts or frequencies of consumption; these can be used when communicating with the public about proposed dietary composition to meet nutritional requirements.

The important goals of the FBDGs and nutrition recommendations include raising the level of nutrition knowledge and nutrition literacy in society. Knowledge of nutrients and nutrition is referred to as nutrition knowledge, which according to some evidence has a positive impact on food-related behaviour but is not alone able to change food habits (4, 5). Nutrition literacy, in turn, is closely related to the concept of health literacy, referring to the ability to obtain, process, understand, and analyse nutrition-related information, including the ability to make sense of food labels (6, 7). Associations with sociodemographic factors are typically detected when assessing the state of nutrition knowledge of populations (5). However, only few studies have examined populations’ nutrition literacy and its relation to sociodemographic determinants (8, 9). Generally, the state of both nutrition knowledge and nutrition literacy and their relationships with either dietary intake or sociodemographic parameters are poorly understood.

For macronutrients, nutrition recommendations suggest rather broad recommended intake ranges that show the interrelationship between macronutrients and allow flexibility in a diet. There is no universal agreement on ideal health-promoting macronutrient intake ranges, but some variation occurs in recommendations given by health authorities around the globe (10). Moreover, different dietary approaches such as low-carbohydrate regimes have own suggestions for macronutrient intakes deviating from those of health authorities (11).

Population adherence to nutrition recommendations is typically monitored by nationwide dietary surveys, which have observed large differences in adherence between macronutrients in Finland (12). For instance, for carbohydrates, only 31% of women and 27% of men met the recommendation and for fibers 21 and 27% accordingly. Around two thirds of both women and men had fat consumption within the recommended range. In turn, adherence to protein recommendation was very high, that is, 85% among women and 77% among men. However, less is known about people’s own perceptions in adherence to nutrition recommendations. Disclosing misperceptions in meeting the recommended intake of macronutrients would potentially help improve the intake of nutrients from being at the insufficient level like fiber in Finland. It has been estimated that premature mortality could be reduced by 25% with shifting from current diets to those recommended in the Nordic Nutrition Recommendations (NNR) (13).

Accordingly, our study aimed to estimate discrepancies between perceived and actual macronutrient intakes and sources in a set of volunteers representing the adult population in Finland. Specifically, we had three goals for this research. First, in relation to the NNR, we compared participants’ perceptions of macronutrient intakes with the actual intakes measured by food records. Second, we compared participants’ perceptions of their main sources of macronutrients with their main actual sources. Third, we examined whether the capacity to self-assess macronutrient intakes was associated with sociodemographic factors.

## Methods

Our study is a cross-sectional sub-study of ScenoProt (Novel protein sources for food security) randomized controlled trial (registered at https://clinicaltrials.gov/ as NCT03206827) conducted at the Department of Food and Nutrition, University of Helsinki, Finland, between December 2016 and June 2017. This sub-study utilized data collected from all participants of the trial at the baseline before they entered the intervention phase and were aware of the assigned intervention group. The original intervention aimed to investigate the effects of replacing animal-source proteins with plant-source proteins on food and nutrient intakes, nutritional status, and biomarkers of chronic diseases in adults.

### Participants and recruitment

Newspapers, mailing lists, the intranet of the University of Helsinki, and Facebook were used to recruit participants aged between 20 and 69 years, with body mass index between 18.5 and 35 kg/m^2^ and willing to follow a randomly assigned intervention diet for 12 weeks. Also, participants were required to be healthy and omnivorous. Exclusion criteria were fasting plasma glucose >6.9 mmol/L and total cholesterol >6.5 mmol/L, inflammatory bowel disease, irritable bowel syndrome, celiac disease, medication for diabetes or hypercholesterolemia, disorders of the endocrine system or lipid metabolism, liver or renal disease, cancer within the past 5 years, regular or recent (in the last 3 months) use of antibiotics, regular use of nutritional supplements, food allergies, eating disorder, strenuous exercising, smoking, pregnancy, or lactation.

Of the 543 interested in participating the trial, 179 (33%) passed the exclusion criteria and clinical screening and of them 145 (81%) consented to participate. As nine people withdrew from the intervention and two did not provide food records, we had 134 (92% of consented) participants with dietary intake data. A paper questionnaire to inquire perceptions of macronutrient intakes was completed by 107 participants, of whom three withdrew from the intervention and two did not provide baseline food records. Consequently, the final number of participants was 102 (70% of consented). A paper questionnaire was used to collect background information and a web-based questionnaire to obtain information on education and employment status. Sample size calculation has been described previously (14).

This study was conducted according to the guidelines laid down in the Declaration of Helsinki and all procedures involving study participants were approved after a full board review by the Coordinating Ethics Committee of the Hospital District of Helsinki and Uusimaa (1651/2016). Written informed consent was obtained from all participants.

### Food records

Actual macronutrient intakes were measured using food records collected at the baseline of the trial with aim to cover 4 days for each participant. Participants were given written and spoken instructions on how to fill in the food records and estimate portion size using household measures and food package labels. Within 1 week, food records were to include 3 weekdays and one weekend day. Only consumed foods and beverages were recorded. Altogether, we had for the analysis 4-day food records from 90 (88%) participants and 2 recording days from one (1%) participant, 3 days from eight (8%) participants, 5 days from two (2%) participants, and 7 days from one (1%) participant.

The food records were examined, and any missing information was requested from the participants. Dietary data were processed using AivoDiet software (version 2.2.0.1, Aivo Oy, Finland) containing Fineli, the National Food Composition Database in Finland (Release 16, Finnish Institute for Health and Welfare, Finland, 2013). A suitable recipe from the database was utilized for each mixed dish, or a new recipe was produced as needed. When necessary, new food items were added to the database. Using the recipes of mixed dishes, food record data were broken down into ingredients before being exported from the dietary software. Thereafter, each ingredient was classified into one of 16 ingredient groups (vegetables; nuts and seeds; legumes; potatoes; fruits and berries; cereals (grains); fat; fish and seafood; meat; egg; milk and dairy products; plant-based dairy substitutes; sugar, confectionery, and chocolate; other products; beverages; and alcoholic beverages) based on Fineli’s classification system (12) with some adjustments such as the addition of the category for plant-based dairy substitutes (Supplementary Tables S1–S2). There were some foods, for example, industrial food products, which were not decomposed into ingredients but were classified in total weight into one of the ingredient groups according to the main ingredient. For example, a biscuit containing both wheat and chocolate was classified into cereals because wheat was the primary ingredient.

### Participants’ perceptions of macronutrient intakes and sources

Our questions, specifically prepared for this study, aimed to identify how well the participants were able to assess their intake of different macronutrients in relation to the official nutrition recommendations (15) and to recognize their main/most important macronutrient sources. Perceptions about macronutrient intakes were investigated by responses to the question: How much carbohydrates/fibers/fats/proteins do you think you get from your current diet? There were six response options for each macronutrient: ‘My intake is well below the recommendation/slightly below the recommendation/according to the recommendation/slightly above the recommendation/well above the recommendation’, and ‘I do not know/cannot answer’. For the analysis, we combined the options ‘well below’ and ‘slightly below’ as one category called ‘below the recommendation’. The options ‘slightly above’ and ‘well above’ were combined as another category called ‘above the recommendation’ except for fiber where we combined the options ‘according to’, ‘slightly above’, and ‘well above’ as one category called ‘according to the recommendation’ since there was no maximum recommended fiber intake given in the NNR as described later. Our aim was to evaluate participants’ genuine perceptions of macronutrient intakes in relation to nutrition recommendations, and therefore, we did not present the recommendations to the participants before the investigation.

To examine participants’ perceptions of the main/most important macronutrient sources in their diets, they were asked an open-ended question for each macronutrient: ‘What are the main/most important sources of carbohydrates/fibers/fats/proteins in your diet?’. The items named by the participants were also classified into 16 ingredient groups (Supplementary Tables S1–S2). One item was one count; however, when the participant named more than one item belonging to the same ingredient group (e.g. milk and cheese), only one of them was counted. A few named items were not eligible to any of the ingredient groups and were omitted from the analysis (Supplementary Tables S1–S2).

### Macronutrient intakes according to the NNR

The NNR proposes a protein intake range of 10–20 E% for adults aged 18–64 years and 15–20 E% for adults aged 65 years and older (1, 15). The recommended carbohydrate intake range is 45–60 E% and fiber intake at least 25–35 g/d for adults, corresponding to approximately 3 g/MJ. The recommended total fat intake range is 25–40 E% for adults, but the intake of saturated fatty acids should be limited to less than 10 E%. Our study was conducted at the time of previous NNR published in 2012 (15), but the recommendations for macronutrient intakes remained essentially unchanged in the update of NNR in 2023 (1).

In the analysis, we had three categories for all macronutrients (intake below/according to/above the recommendation) except two for fiber (intake below/according to the recommendation). We used one decimal place for thresholds, applying the rounding rule that if the first digit to be omitted is five or more, the last digit remaining is increased by one. Consequently, thresholds for, for instance, categories of protein in age group 18–64 years were ‘below the recommendation ≤9.4 E%’, ‘according to the recommendation 9.5–20.4 E%’, and ‘above the recommendation ≥20.5 E%’.

### Statistical analyses

Descriptive analysis for participant characteristics were conducted including frequencies or means with standard deviations (SD). Considering a participant’s whole food record period, the mean daily intake of each macronutrient was estimated in E%. We calculated the frequencies of participants with different perceptions of their macronutrient intakes. The proportions of participants in each category based on both perceived and actual intake were calculated. In each calculation, we assumed a binomial distribution and used the Wilson score interval method to estimate a binomial proportion confidence interval with a 95% confidence level.

We created a binary variable called the capacity to self-assess macronutrient intakes in relation to the official nutrition recommendations. First, we counted how often the participant’s perceived macronutrient intake and actual intake were aligned. Second, considering each macronutrient separately, we gave one count for the incidence when the category of the participant’s perceived intake and the category of actual intake were the same and zero count otherwise. Third, we summed up the counts; each participant had a total count between zero and four, and this was further categorized into a binary variable of 0–2 counts, reflecting lower self-assessment capacity, and 3–4 counts reflecting higher self-assessment capacity. Next, we investigated associations between sociodemographic determinants and the capacity to self-assess macronutrient intakes in relation to the official nutrition recommendations by logistic regression analysis using the binary variable as an outcome. Odd ratios with 95% confidence intervals were calculated for categorized age, gender, and education in a crude model and in an adjusted model where all variables were included. The reference category for each variable was the category with lowest frequency of having 3–4 counts (i.e. gender: male; education: high school level or lower; age: 60–69 years), and each of the remaining categories was compared with the reference. As education data were lacking from eight participants, we incorporated 94 individuals in the adjusted model.

For each macronutrient, the mean daily intake (in grams) from each ingredient group as a percentage of total mean daily intake (in grams) was computed. Further, a frequency of each ingredient group as the main/most important macronutrient source was calculated for each macronutrient as a percentage based on the baseline questionnaire data. Statistical analyses were performed using SPSS Statistics for Windows versions 27 & 29 (IBM Corp., Armonk, NY, USA).

## Results

The characteristics of the study population with a mean age of 47 (SD 15) years are shown in Table 1. The majority (78%) of participants were women. Data on education level were available for 92% of participants, more than three-quarters of whom had at least a bachelor’s degree. Among all participants, the mean daily intakes (E%) of protein and fat were within the recommended intake range (Table 1). In contrast, the mean daily carbohydrate intake (E%) was below the recommended intake range. The mean daily fiber intake (both in g and g/MJ) exceeded the minimum recommended intake.

**Table 1:** Participant characteristics and mean daily energy and nutrient intakes in the ScenoProt trial (*n* = 102)

Characteristics	Mean (SD) or *n* (%)
Age, years, mean (SD)	47.1 (14.8)
Categorized age, *n* (%)	
20–29	17 (17)
30–39	19 (19)
40–49	18 (18)
50–59	22 (22)
60–69	26 (25)
Gender, *n* (%)	
Female	80 (78)
Male	22 (22)
BMI, kg/m^2^, mean (SD)	24.8 (4.0)
Energy intake, MJ, mean (SD)	
Female (*n* = 80)	8.3 (1.4)
Male (*n* = 22)	10.9 (2.2)
Energy intake, kcal, mean (SD)	
Female (*n* = 80)	1,988 (344)
Male (*n* = 22)	2,605 (531)
Carbohydrate intake, E%^a^, mean (SD)	40.3 (6.1)
Fiber intake, g, mean (SD)	29.1 (9.8)
Fiber intake, g/MJ^a^, mean (SD)	3.4 (0.9)
Fat intake, E%^a^, mean (SD)	38.0 (5.8)
Saturated fatty acid intake, E%^a^, mean (SD)	13.0 (3.4)
Protein intake, E%^a^, mean (SD)	
All participants	18.5 (3.4)
Participants aged 20–64 years (*n* = 86)	18.7 (3.6)
Participants aged 65–69 years (*n* = 16)	17.0 (2.0)
Education^b^, *n* (%)	
High school level or lower	22 (23)
Bachelor’s degree or equivalent	29 (31)
Master’s degree or higher	43 (46)
Employment^b^, *n* (%)	
Employed/partly employed	68 (72)
Not employed	26 (28)

E%, a proportion from the total amount of energy.

a Alcohol excluded from energy.

b Data available for 94 (92%) participants.

**Fig. 1:**
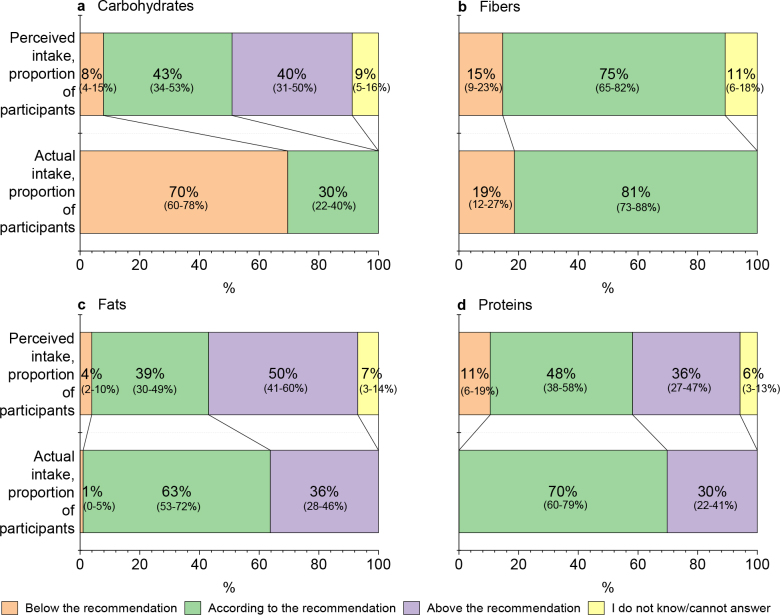
Proportions of participants with different perceptions of their (a) carbohydrate, (b) fiber, (c) fat, and (d) protein intakes versus proportions of participants with different actual mean daily intakes in relation to the Nordic Nutrition Recommendations in the ScenoProt trial. Confidence intervals in parentheses, confidence level 95%. *n* = 86 for the protein intake (participants aged 20–64 years), otherwise *n* = 102 (all participants including those ≥ 65 years old).

Participants’ perceptions of their macronutrient intakes deviated from the actual mean daily intakes (E%), except for fiber, measured in relation to the NNR (Fig. 1). The biggest differences between perception and actual mean daily intake were regarding carbohydrates. More than 80% of participants believed they received carbohydrates in line with or above the recommendation, while their actual mean daily carbohydrate intake was below the recommended intake range for 70%, and no one exceeded the recommended range (Fig. 1; Supplementary Table S3). Three-quarters of the participants thought that their fiber intake was at the recommended level, which corresponded well with the result measured by food records (Fig. 1; Supplementary Table S4). Regarding fats, nearly two-thirds of participants met the recommended intake range and over one-third exceeded it (Fig. 1; Supplementary Table S5). However, when measuring perceptions, the opposite was true, that is, the share of participants who thought that their fat intake exceeds the recommendation was larger than the share of those who believed that they were within the recommended range. Of the participants aged 20–64 years, 70% met the recommended protein intake range and the rest exceeded it (Fig. 1; Supplementary Table S6). Still, over one-tenth of participants perceived their protein intake to be lower than the recommended intake range. The share of those who answered ‘I do not know/cannot answer’ varied between 6 and 11%, the percentage being lowest for protein intake and highest for fiber intake (Fig. 1; Supplementary Tables S3–S6).

Among 21% of participants, the self-estimated intake and actual mean intake in relation to the nutrition recommendations fell into the same category for at least three macronutrients (the range from 0 to 4 macronutrients) (Supplementary Table S7). In our sample, no statistically significant associations emerged between age, gender, or education and the capacity to self-assess macronutrient intakes in relation to the official nutrition recommendations (Table 2).

**Table 2:** Associations between sociodemographic determinants and the higher capacity to self-assess macronutrient intakes in relation to the Nordic Nutrition Recommendations^a^ in the ScenoProt trial

Sociodemographic variables	Crude			Adjusted^b^		
	OR	95% CI	*P* ^c^	OR	95% CI	*P* ^c^
Age category, reference: 60–69 years (*n* = 102)			0.481			0.437
20–29 years	3.69	0.59–22.94	0.161	3.57	0.51–25.14	0.201
30–39 years	3.20	0.52–19.67	0.209	2.49	0.36–17.32	0.358
40–49 years	4.62	0.78–27.19	0.091	4.64	0.75–28.71	0.099
50–59 years	4.50	0.81–25.15	0.087	4.90	0.83–28.84	0.079
Gender, reference: Male (*n* = 102)						
Female	1.21	0.36–4.07	0.753	1.43	0.36–5.72	0.616
Education, reference: High school level or lower (*n* = 94)			0.662			0.759
Bachelor’s degree or equivalent	1.65	0.36–7.50	0.515	1.32	0.26–6.76	0.740
Master’s degree or higher	1.92	0.47–7.85	0.364	1.75	0.38–7.96	0.472

a The outcome variable (the capacity to self-assess macronutrient intake in relation to the official nutrition recommendations) was created in following steps: 1) Counting of incidences when the participant’s perceived macronutrient intake and the actual intake were aligned; 2) Considering each macronutrient separately, giving one count for the incidence when the category of the participant’s perceived intake and the category of actual intake were aligned and zero count otherwise; 3) Summing up the counts, when each participant had a total count between 0 and 4 (Supplementary Table S7), which was further categorized into a binary variable, that is, 0–2 counts and 3–4 counts; 4) The higher capacity, that is, 3–4 counts was used as the reference outcome in the logistic regression analysis.

b Adjusted for the remaining variables (*n* = 94). Log likelihood 88.669; Cox & Snell R Square 0.061; Nagelkerke *R* Square 0.097.

c *P* value for the association between the variable and the higher capacity to self-assess macronutrient intakes. Within the category, *P*-values for comparison to the reference.

**Fig. 2:**
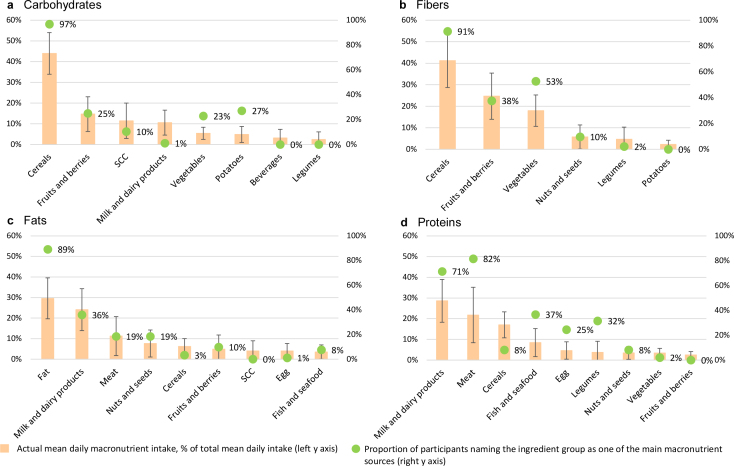
Participants’ perceptions of main macronutrient sources in their diets (marked with circles, right y axis) versus actual macronutrient sources (shown in bars with standard deviation, left y axis) by ingredient group in the ScenoProt trial (*n* = 102). For (a) carbohydrate, the key ingredient groups are presented using minimum 2% of total actual mean daily intake as the threshold for ingredient groups; 6% of the participants did not name any sources. For (b) fiber, the key ingredient groups are presented using minimum 2% of total actual mean daily intake as the threshold for ingredient groups; 9% of the participants did not name any sources. For (c) fat, the key ingredient groups are presented using minimum 4% of total actual mean daily intake as the threshold for the ingredient groups; 10% of the participants did not name any sources. For (d) protein, the key ingredient groups are presented using minimum 2% of total actual mean daily intake as the threshold for ingredient groups; 4% of the participants did not name any sources. SCC, sugar, confectionery, and chocolate.

Cereals, which nearly all participants named as one of the main carbohydrate sources, covered 44% of the mean daily carbohydrate intake (Fig. 2; Table 3). Potatoes and vegetables were listed more frequently as the main carbohydrate sources than the two other ingredient groups, namely sugar, confectionery, and chocolate and milk and dairy products, which was named only by one participant. However, the latter groups contributed on average each 11% to the total mean daily carbohydrate intake, while the former groups each contributed only 5%. The participants’ perceptions of the main fiber sources corresponded well with the measured main fiber sources (Fig. 2; Table 4). The main sources of fat were quite well recognized by the participants, except for cereals and eggs, noted by a few participants, and sugar, confectionery, and chocolate noted by no participants as one of the main fat sources despite cereals contributing to the mean daily fat intake by 6% and other two groups both by 4% (Fig. 2; Table 5). Regarding the main protein sources, the participants rarely cited cereals although this ingredient group was on average the third largest source of protein after milk and dairy products and meat (Fig. 2; Table 6). Nearly one-third of participants named legumes as one of the main protein sources, but less than 4% of the total mean daily protein intake was derived from legumes.

**Table 3:** Participants’ perceptions of their main/most important carbohydrate sources in their diets versus actual carbohydrate sources measured by food records in the ScenoProt trial (*n* = 102)

Ingredient group	Frequency of participants who named an ingredient from the group as one of their main carbohydrate sources	Actual carbohydrate intake from the ingredient group
	*n* (%)^a^	Mean (SD), % of total daily carbohydrate intake
Cereals	93 (97)	43.9 (10.1)
Fruits and berries	24 (25)	14.7 (8.4)
Sugar, confectionery, and chocolate	10 (10)	11.4 (8.5)
Milk and dairy products (including sour milk products, cheese, and other milk products, e.g., whey powder)	1 (1)	10.5 (6.0)
Vegetables	22 (23)	5.4 (2.9)
Potatoes	26 (27)	4.8 (3.8)
Beverages	0 (0)	3.1 (4.2)
Legumes	0 (0)	2.4 (3.6)
Plant-based dairy substitutes	0 (0)	0.8 (1.9)
Other products	0 (0)	0.8 (1.3)
Nuts and seeds	0 (0)	0.7 (0.8)
Alcoholic beverages	0 (0)	0.7 (1.4)
Meat (including sausages and meat products)	0 (0)	0.4 (0.8)
Fat (oil, margarine and vegetable fat spreads, butter, fat blends, and other fats, e.g., dressings, tallow, lard)	0 (0)	0.2 (0.2)
Egg	0 (0)	0.1 (0.1)
Fish and seafood	0 (0)	0.0 (0.1)

a Of all participants, 6 (6%) did not name any carbohydrate sources in their diets.

**Table 4:** Participants’ perceptions of their main/most important fiber sources in their diets versus actual fiber sources measured by food records in the ScenoProt trial (*n* = 102)

Ingredient group	Frequency of participants who named an ingredient from the group as one of their main fiber sources	Actual fiber intake from the ingredient group
	*n* (%)^a^	Mean (SD), % of total daily fiber intake
Cereals	85 (91)	41.2 (12.5)
Fruits and berries	35 (38)	24.7 (10.7)
Vegetables	49 (53)	18.0 (7.3)
Nuts and seeds	9 (10)	5.8 (5.6)
Legumes	2 (2.2)	4.6 (5.7)
Potatoes	0 (0)	2.3 (1.9)
Sugar, confectionery, and chocolate	0 (0)	1.4 (1.8)
Plant-based dairy substitutes	0 (0)	0.8 (1.6)
Other products	0 (0)	0.7 (0.8)
Milk and dairy products (including sour milk products, cheese, and other milk products, e.g., whey powder)	0 (0)	0.3 (0.7)
Meat (including sausages and meat products)	0 (0)	0.2 (0.7)
Fat (oil, margarine and vegetable fat spreads, butter, fat blends, and other fats, e.g., dressings, tallow, lard)	0 (0)	0.0 (0.1)
Beverages	0 (0)	0.0 (0.1)
Fish and seafood	0 (0)	0.0 (0.0)
Egg	0 (0)	0.0 (0.0)
Alcoholic beverages	0 (0)	0.0 (0.0)

a Of all participants 9 (9%) did not name any fiber sources in their diets

**Table 5:** Participants’ perceptions of their main/most important fat sources in their diets versus actual fat sources measured by food records in the ScenoProt trial (*n* = 102)

Ingredient group	Frequency of participants who named an ingredient from the group as one of their main fat sources	Actual fat intake from the ingredient group
	*n* (%)^a^	Mean (SD), % of total daily fat intake
Fat (oil, margarine and vegetable fat spreads, butter, fat blends, and other fats, e.g., dressings, tallow, lard)	82 (89)	29.6 (10.0)
Milk and dairy products (including sour milk products, cheese, and other milk products, e.g., whey powder)	33 (36)	24.1 (10.2)
Meat (including sausages and meat products)	17 (18)	11.2 (9.5)
Nuts and seeds	17 (18)	7.6 (6.6)
Cereals	3 (3)	6.2 (3.8)
Fruits and berries	9 (10)	4.8 (6.9)
Sugar, confectionery, and chocolate	0 (0)	4.0 (4.9)
Egg	1 (1)	4.0 (3.7)
Fish and seafood	7 (8)	3.5 (3.5)
Vegetables	0 (0)	1.6 (1.9)
Plant-based dairy substitutes	0 (0)	1.1 (2.2)
Legumes	0 (0)	0.9 (1.6)
Beverages	0 (0)	0.5 (0.4)
Potatoes	0 (0)	0.5 (1.3)
Other products	2 (2)	0.3 (0.6)
Alcoholic beverages	0 (0)	0.0 (0.0)

a Of all participants, 10 (10%) did not name any fat sources in their diets.

**Table 6:** Participants’ perceptions of their main/most important protein sources in their diets versus actual protein sources measured by food records in the ScenoProt trial (*n* = 102)

Ingredient group	Frequency of participants who named an ingredient from the group as one of their main protein sources	Actual protein intake from the ingredient group
	*n* (%)^a^	Mean (SD), % of total daily protein intake
Milk and dairy products (including sour milk products, cheese, and other milk products, e.g., whey powder)	70 (71)	28.6 (10.3)
Meat (including sausages and meat products)	80 (82)	21.8 (13.4)
Cereals	8 (8)	17.1 (6.3)
Fish and seafood	36 (37)	8.5 (6.8)
Egg	24 (24)	4.6 (4.2)
Legumes	31 (32)	3.7 (5.4)
Nuts and seeds	8 (8)	3.5 (3.1)
Vegetables	2 (2)	3.4 (2.3)
Fruits and berries	0 (0)	2.5 (1.6)
Beverages	0 (0)	1.6 (0.9)
Sugar, confectionery, and chocolate	0 (0)	1.3 (1.9)
Potatoes	0 (0)	1.3 (1.0)
Plant-based dairy substitutes	0 (0)	0.8 (1.8)
Other products	2 (2)	0.7 (1.6)
Alcoholic beverages	0 (0)	0.3 (0.5)
Fat (oil, margarine and vegetable fat spreads, butter, fat blends, and other fats, e.g., dressings, tallow, lard)	0 (0)	0.2 (0.2)

a Of all participants, 4 (4%) did not name any protein sources in their diets.

## Discussion

Reflected in the light of the NNR, we evaluated differences between perceived and actual macronutrient intakes among healthy working-age Finnish adults using baseline food records from a dietary trial. Furthermore, we examined how well participants’ perceptions of macronutrient sources corresponded to the actual sources. Between perceived and actual macronutrient intakes, we found a pronounced discrepancy for carbohydrates, but gaps existed also for fats and proteins. Concerning sources of macronutrients, some misperceptions were revealed. This is the only study of which we are aware that examined perceived macronutrient intakes and sources against actual intakes and sources using the official nutrition recommendations as a reference.

The most distinct gap emerged between perceived carbohydrate intake and real intake, where 40% of the participants thought that they had consumed more carbohydrates than stated in the nutrition recommendations, while actually more than two-thirds consumed less than recommended. Similar results on the actual carbohydrate intake have been obtained from the national dietary survey in Finland (FinDiet), where 69% of women and 73% of men had lower carbohydrate intake than stated in the recommendation (12). The participants who assumed that they consumed more carbohydrates than recommended may have had a misconception that the nutrition recommendations support fairly low carbohydrate consumption. Consequently, because they thought that their carbohydrate intake exceeded the recommendation, they may have actively reduced their intake, resulting in an eventually lower consumption of carbohydrates than recommended. However, divergencies in perceived carbohydrate intake were not reflected in energy intake and same was true when differences in actual carbohydrate intake were considered (data not shown). In women, average energy intake aligned with reference levels for a sedentary physical lifestyle, while men’s energy intake corresponded to recommended levels for moderate physical activity (1). Not just the amount but the quality of carbohydrates is emphasized in the nutrition recommendations, which encourage the consumption of fiber-rich foods such as whole grains over refined grains. Erroneous interpretations of carbohydrate intake can result in an unbalanced diet and an increased risk of several noncommunicable diseases. In Nordic countries, diets low in whole grains were identified as the primary contributors to ischemic heart disease and colon and rectal cancer (1).

The large share of participants consuming carbohydrates below the recommended range might be related to the carbohydrate-conscious eating that emerged in Finland in the early 2000s but gained widespread popularity only in 2011 (16). In 2012, about 7% of Finnish adults stated that they followed a low-carbohydrate diet (LCD) (17). LCDs are being marketed for efficient weight loss, but both their short-term and long-term outcomes are like those of diets with a balanced macronutrient composition and aimed at weight reduction (18). The movement supporting LCDs has been characterized by challenging the evidence behind the established recommendations associating saturated fat consumption with high blood cholesterol and calling for more personalized approaches in the design of nutrition recommendations (19). On the contrary, people who do not follow LCDs more likely comply with dietary guidelines and have more knowledge about carbohydrates (20).

Almost all participants named cereals as one of their main carbohydrate sources, which is obvious as cereals are in general the most common carbohydrate source in human nutrition. In turn, the participants did not know that milk and dairy products, which naturally include lactose, were a key source of carbohydrates. Of macronutrients, potatoes mainly provide carbohydrates, but their contribution to the total carbohydrate intake may be lower than people generally expect. That was shown in our study, where potatoes were not a prominent source of carbohydrates but were assumed to be so by some participants. The sugar, confectionery, and chocolate ingredient group were less frequently named as one of the main carbohydrate sources by participants. This was not surprising as these palatable foods are not typically consumed to merely satisfy energy and nutrient needs but are eaten for their hedonic properties (21).

Regarding fibers, we found that the perceived and actual intakes and sources were aligned, and the participants followed a fiber-rich diet. Over 80% of our participants, but only 21% of women and 27% of men in the FinDiet survey (12), reached the recommended fiber intake. This finding proves that even though the energy received from carbohydrates was lower than suggested, the nutritional quality of carbohydrates was high as they included plenty of fibers. An ideal daily diet includes different fiber sources to ensure as versatile and plentiful fiber intake as possible. In our study, besides the consumption of cereals, fruits, berries, and vegetables, the high and varied fiber intake was derived from other fiber dense foods namely nuts, seeds, and legumes. In an effort to improve fiber intake at the population level, FBDGs could emphasize the importance of all the above food groups for the sufficient fiber intake.

Half of the participants thought that they had exceeded the recommended fat intake range, even though the largest proportion of participants remained within the recommendation. In our study, compared with the FinDiet survey (12), a slightly higher share of participants consumed more fat than recommended. Furthermore, fat products and meat had clearly smaller shares in total fat intake, and fat intake was more evenly distributed among different ingredient groups than in the Finnish national dietary survey. Our participants were aware of the main fat sources in their diets, excluding cereals and eggs. The egg result can be considered surprising, as in general egg yolk is a well-known source of fat. An application of carbohydrate-conscious eating is a low-carbohydrate high-fat diet that promotes the use of sources of saturated fat such as butter (19). Our results gave the impression that people are consciously investing in their fat intake. Actually, we found that more than 80% of participants got more saturated fatty acids than suggested by the NNR, which emphasize the use of unsaturated fatty acid sources.

Nearly half of the participants believed that their protein intake was either below or above the recommendation, while the actual intake was within the recommended range for most participants. However, our sample was characterized by abundant protein intake that was even higher than in the FinDiet survey (12). Followed by milk and dairy products and meat, cereals finished in the top three most common actual sources of proteins, but this fact was not well known by participants. This outcome is unsurprising since the role of cereals as a protein source in mixed diets is not usually highlighted. Third of the participants had the impression that legumes were one of the most important sources of protein in their diets even though the share of legumes in the total protein intake was quite low. However, protein intake from legumes, nuts, and seeds combined was slightly higher and that from meat slightly lower in this study than in the Finnish adult population in general.

Previously, participants’ perceived dietary quality has been compared with their adherence to FBDGs (22) and with dietary index scores (23). Among athletes, perceived macronutrient intakes have been compared with actual intakes and the official dietary guidelines for athletes (24). Furthermore, an earlier study stated that the nutrition knowledge of Finnish adolescent athletes and their coaches requires improvement (25). Nutrition knowledge has been positively associated with age, female gender, education, and socioeconomic status (5). Also, nutrition literacy has been positively associated with sociodemographic factors such as age, education, income, and work skill level (8, 9). Contrarily, we did not detect associations between age, gender, or education and the capacity to self-assess macronutrient intakes that we assume might be due to the lack of diversity in terms of gender and education levels in our sample. Considerable discrepancies existed between perceived and actual food consumption patterns in relation to the nutrition recommendations though good nutrition knowledge and nutrition literacy could have been expected in our highly educated and female-dominated sample. Consequently, we may assume that gaps between the perceived and actual food intakes would have been even more distinct had they been investigated in the general population.

Lack of nutrition knowledge and nutrition literacy could be a barrier to follow the nutrition recommendations and FBDGs. While we did not directly explore either nutrition knowledge or nutrition literacy, participants’ perceptions of macronutrient intakes were evaluated. However, macronutrients and related recommendations may be difficult concepts for people to grasp. Compared with the recommendations for single nutrients, FBDGs are likely easier to comprehend by the public. Still, the scientific evidence is quite poor in terms of people’s awareness of FBDGs, and whether they understand the information provided in them or use them in making food choices (26). This kind of data is crucial in assessing the effectiveness of these guidelines in relation to their targets such as modifying food-related behaviour in a healthier direction.

In addition to public bodies, companies have shown interest in guiding consumers to make choices in accordance with the nutrition recommendations. For this purpose, the largest grocery chains in Finland have launched digital applications for the use of their loyalty card customers (27, 28). Through these applications, consumers can monitor how well their food purchases at shopping cart level comply with the national nutrition recommendations in terms of food consumption and nutrient intake. Recently, it was demonstrated that consumers increased fruit and vegetable purchases after using feedback provided the digital application (29). Consequently, these applications may be considered potential tools to guide food habits of their users towards the official nutrition recommendations. Also, it has been shown that grocery purchases are able to moderately characterize food consumption at the population level (30).

Strengths of the study include that we measured macronutrient intakes by food records giving more accurate estimates compared to food frequency questionnaires. Also, the meticulous data processing covering a disaggregation of dishes from food records into ingredients and a classification of these into ingredient groups was a strength of this study. A limitation was that the questionnaire to inquire perceptions of nutrient intakes was not previously tested but used first time for scientific purposes and could be developed based on the experiences from this study. For instance, using multiple-choice questions instead of open-ended questions to inquire about the main macronutrient sources in participants’ diets could have influenced the outcome as participants might fail to recall some food items when answering the open-ended questions. Regarding the external validity of the findings, we might suppose that if the disparity between perceived and actual nutrient intakes had been explored in the general population, it would have been even more pronounced than in our highly educated sample including mostly women. Moreover, the average diet followed by the participants had many characteristics related to healthy diets, such as high fiber content, that might reflect the participants being more health-conscious than people in general. Also, the participants were intended to participate in the 3-month intervention focusing on sustainable eating and examining the health effects of diets with a varying mix of proteins from both plant-based and animal-based sources, which in turn might reflect their having more interest in diets and health than the general population.

Evidence is scarce for this topic, and new perspectives for research are warranted. In the future, the reasoning behind the misconceptions between perceived and actual macronutrient intakes related to the nutrition recommendations should be elucidated. Also, the state of nutrition knowledge and nutrition literacy among different populations requires further clarification. Of great importance is to determine how people’s perceptions of dietary intake and level of nutrition knowledge and nutrition literacy affect food-related behaviour.

Moving towards a healthier diet is likely to be more challenging for individuals who have a misperception about their macronutrient intake (31, 32). Consequently, when promoting healthy and sustainable diets and developing interventions targeted at modifying food habits, it is important to bear in mind that a discrepancy between subjective and objective estimates of dietary intake can be an obstacle to dietary change. In other words, having a realistic picture of one’s own diet might facilitate the dietary transition.

## Conclusion

We noted a discrepancy between perceived macronutrient consumption patterns and actual patterns measured in relation to the nutrition recommendations in the highly educated and female-dominated population. The most distinct gap emerged between the perceived carbohydrate intake and the actual intake, but gaps existed also in fat and protein intakes. Cereals were one of the most prevalent protein sources; however, the participants were unaware of this. Our research proposes that self-assessment of macronutrient consumption in relation to the nutrition recommendations is challenging. Misperceptions about dietary intake could be potentially reduced by finding more informative and instructive ways to disseminate FBDGs and nutrition recommendations to the public.

## Supplementary Material



## References

[1] Blomhoff R, Andersen R, Arnesen EK, Christensen JJ, Eneroth H, Erkkola M, et al. Nordic Nutrition Recommendations 2023. Copenhagen: Nordisk Ministerråd; 2023. doi: 10.6027/nord2023-003

[2] Jahns L, Davis-Shaw W, Lichtenstein AH, Murphy SP, Conrad Z, Nielsen F. The history and future of dietary guidance in America. Adv Nutr 2018; 9(2): 136–47. doi: 10.1093/advances/nmx025 29659693 PMC5916427

[3] Fogelholm M. Nutrition recommendations and science: next parallel steps. J Sci Food Agric 2016; 96(4): 1059–63. doi: 10.1002/jsfa.7479 26531226

[4] Worsley A. Nutrition knowledge and food consumption: can nutrition knowledge change food behaviour? Asia Pac J Clin Nutr 2002; 11: S579–85. doi: 10.1046/j.1440-6047.11.supp3.7.x 12492651

[5] Barbosa LB, Vasconcelos SML, Correia LO dos S, Ferreira RC. Nutrition knowledge assessment studies in adults: a systematic review. Cien Saude Colet 2016; 21(2): 449–62. doi: 10.1590/1413-81232015212.20182014 26910153

[6] Krause C, Sommerhalder K, Beer-Borst S, Abel T. Just a subtle difference? Findings from a systematic review on definitions of nutrition literacy and food literacy. Health Promot Int 2018; 33(3): 378–89. doi: 10.1093/heapro/daw084 27803197 PMC6005107

[7] Vettori V, Lorini C, Milani C, Bonaccorsi G. Towards the implementation of a conceptual framework of food and nutrition literacy: providing healthy eating for the population. Int J Environ Res Public Health 2019; 16(24): 5041. doi: 10.3390/ijerph16245041 31835678 PMC6950737

[8] Michou M, Panagiotakos DB, Lionis C, Costarelli V. Socioeconomic inequalities in relation to health and nutrition literacy in Greece. Int J Food Sci Nutr 2019; 70(8): 1007–13. doi: 10.1080/09637486.2019.1593951 30935258

[9] Gibbs HD, Kennett AR, Kerling EH, Yu Q, Gajewski B, Ptomey LT, et al. Assessing the nutrition literacy of parents and its relationship with child diet quality. J Nutr Educ Behav 2016; 48(7): 505–509.e1. doi: 10.1016/j.jneb.2016.04.006 27216751 PMC4931947

[10] Venn B. Macronutrients and human health for the 21st century. Nutrients 2020; 12(8): 2363. doi: 10.3390/nu12082363 32784664 PMC7468865

[11] Churuangsuk C, Kherouf M, Combet E, Lean M. Low-carbohydrate diets for overweight and obesity: a systematic review of the systematic reviews. Obes Rev 2018; 19(12): 1700–18. doi: 10.1111/obr.12744 30194696

[12] Valsta L, Kaartinen N, Tapanainen H, Männistö S, Sääksjärvi K, editors. Ravitsemus Suomessa – FinRavinto 2017 – tutkimus (Nutrition in Finland – The National FinDiet 2017 Survey). Helsinki: Finnish institute for health and welfare; 2018. Available from: https://urn.fi/URN:ISBN:978-952-343-238-3 [cited 16 December 2024].

[13] Wood A, Gordon LJ, Röös E, Karlsson JO, Häyhä T, Bignet V, et al. Nordic food systems for improved health and sustainability: baseline assessment to inform transformation. Stockholm: Stockholm Resilience Centre; 2019. Available from: https://www.stockholmresilience.org/download/18.66e0efc517643c2b810381d/1618468740819/SRC_Report Nordic Food Systems_ June 2019 adapted.pdf [cited 16 December 2024].

[14] Päivärinta E, Itkonen ST, Pellinen T, Lehtovirta M, Erkkola M, Pajari AM. Replacing animal-based proteins with plant-based proteins changes the composition of a whole Nordic diet – a randomised clinical trial in healthy Finnish adults. Nutrients 2020; 12(4): 943. doi: 10.3390/nu12040943 32231103 PMC7231027

[15] Nordic Council of Ministers. Nordic Nutrition Recommendations 2012: integrating nutrition and physical activity. Copenhagen: Nordisk Ministerråd; 2014. doi: 10.6027/Nord2014-002

[16] Jauho M, Pääkkönen J, Isotalo V, Pöyry E, Laaksonen SM. How do trendy diets emerge? An exploratory social media study on the low-carbohydrate diet in Finland. Food Cult Soc 2021; 26(2): 344–69. doi: 10.1080/15528014.2021.1971436

[17] Jallinoja P, Niva M, Helakorpi S, Kahma N. Food choices, perceptions of healthiness, and eating motives of self-identified followers of a low-carbohydrate diet. Food Nutr Res 2014; 58: 23552. doi: 10.3402/fnr.v58.23552 25490960 PMC4258637

[18] Naude CE, Brand A, Schoonees A, Nguyen KA, Chaplin M, Volmink J. Low-carbohydrate versus balanced-carbohydrate diets for reducing weight and cardiovascular risk. Cochrane Database Syst Rev 2022; 1(1): CD013334. doi: 10.1002/14651858.CD013334.pub2 35088407 PMC8795871

[19] Jauho M. The social construction of competence: conceptions of science and expertise among proponents of the low-carbohydrate high-fat diet in Finland. Public Underst Sci 2016; 25(3): 332–45. doi: 10.1177/0963662514558167 25415233

[20] Churuangsuk C, Lean MEJ, Combet E. Carbohydrate knowledge, dietary guideline awareness, motivations and beliefs underlying low-carbohydrate dietary behaviours. Sci Rep 2020; 10: 14423. doi: 10.1038/s41598-020-70905-2 32879368 PMC7468104

[21] Levine AS, Kotz CM, Gosnell BA. Sugars: hedonic aspects, neuroregulation, and energy balance. Am J Clin Nutr 2003; 78(4): 834S–42S. doi: 10.1093/ajcn/78.4.834S 14522747

[22] Batis C, Castellanos-Gutiérrez A, Aburto TC, Jiménez-Aguilar A, Rivera JA, Ramírez-Silva I. Self-perception of dietary quality and adherence to food groups dietary recommendations among Mexican adults. Nutr J 2020; 19(59). doi: 10.1186/s12937-020-00573-5 PMC7310597 32571341

[23] Powell-Wiley TM, Miller PE, Agyemang P, Agurs-Collins T, Reedy J. Perceived and objective diet quality in US adults: a cross-sectional analysis of the National Health and Nutrition Examination Survey (NHANES). Public Health Nutr 2014; 17(12): 2641–9. doi: 10.1017/S1368980014000196 24636343 PMC4190093

[24] Jagim AR, Zabriskie H, Currier B, Harty PS, Stecker R, Kerksick CM. Nutrient Status and perceptions of energy and macronutrient intake in a Group of Collegiate Female Lacrosse Athletes. J Int Soc Sports Nutr 2019; 16(43). doi: 10.1186/s12970-019-0314-7 PMC6794738 31615510

[25] Heikkilä M, Valve R, Lehtovirta M, Fogelholm M. Nutrition knowledge among young Finnish endurance athletes and their coaches. Int J Sport Nutr Exerc Metab 2018; 28(5): 522–7. doi: 10.1123/ijsnem.2017-0264 29252046

[26] Brown KA, Timotijevic L, Barnett J, Shepherd R, Lähteenmäki L, Raats MM. A review of consumer awareness, understanding and use of food-based dietary guidelines. Br J Nutr 2011; 106(1): 15–26. doi: 10.1017/S0007114511000250 21385508

[27] S Group. S Group launches a personal trainer for grocery shopping. 2020. Available from: https://s-ryhma.fi/en/news/s-group-launches-a-personal-trainer-for-grocery-sh/55hyMEuot99OX3JhxRaSHd [cited 16 December 2024].

[28] K Group. K Group introduces new service: K-Ostokset now shows the nutritional values of your grocery purchases. 2020. Available from: https://www.kesko.fi/en/media/news-and-releases/news/2020/k-group-introduces-new-service-k-ostokset-now-shows-the-nutritional-values-of-your-grocery-purchases/ [cited 16 December 2024].

[29] Koski H, Kuikkaniemi K, Pantzar M. Do grocery feedback systems enabling access to past consumption impact individual food purchase behavior? ETLA Working Papers No 103. Helsinki: ETLA Economic Research; 2023. Available from: https://www.etla.fi/wp-content/uploads/Etla-Working-Paper-103.pdf [cited 16 December 2024].

[30] Vepsäläinen H, Nevalainen J, Kinnunen S, Itkonen ST, Meinilä J, Männistö S, et al. Do we eat what we buy? Relative validity of grocery purchase data as an indicator of food consumption in the LoCard study. Br J Nutr 2022; 128(9): 1780–8. doi: 10.1017/s0007114521004177 34657639

[31] de Menezes MC, Mingoti SA, Mendonça RdD, Lopes ACS. Mistaken perception of lipid intake and its effects: a randomized trial. BMC Nutr 2017; 3(1): 77. doi: 10.1186/s40795-017-0193-8 32153854 PMC7050849

[32] Paisley C, Lloyd H, Sparks P, Mela DJ. Consumer perceptions of dietary changes for reducing fat intake. Nutr Res 1995; 15(12): 1755–66. doi: 10.1016/0271-5317(95)02045-4

