# Supplementary material for: Identifying gaps between perceived and actual intakes in Finnish adults: self-assessment of macronutrient intakes in relation to nutrition recommendations is challenging
Source: Food Nutr Res. 2025 Feb 4;69:10.29219/fnr.v69.10535. doi: 10.29219/fnr.v69.10535 (PMC12509224; doi:10.29219/fnr.v69.10535)
Supplement: Supplementary file 1 [file FNR-69-10535-s1.pdf]

Supplementary material

Journal name

Food & Nutrition Research

Article title

Identifying gaps between perceived and actual intakes in Finnish adults: self-assessment of macronutrient intakes in relation to nutrition recommendations is challenging

Authors

Sari Bäck, Tiina Pellinen, Essi Päivärinta, Petra Rautio, Antti Isokangas, Maijaliisa Erkkola, Anne-Maria Pajari

Corresponding author

Anne-Maria Pajari, E-mail: anne-maria.pajari@helsinki.fi

University of Helsinki, Department of Food and Nutrition, Agnes Sjöbergin katu 2, FI-00014 University of Helsinki, Helsinki, Finland

Contents

Table S1 Carbohydrate and fiber sources named by participants and ingredient groups where carbohydrate sources were included in the ScenoProt trial

Table S2 Fat and protein sources named by participants and ingredient groups where fat sources were included in the ScenoProt trial

Table S3 Participants' perceptions of their carbohydrate intake versus actual carbohydrate intake in relation to the Nordic Nutrition Recommendations in the ScenoProt trial

Table S4 Participants' perceptions of their fiber intake versus actual fiber intake in relation to the Nordic Nutrition Recommendations in the ScenoProt trial

Table S5 Participants' perceptions of their fat intake versus actual fat intake in relation to the Nordic Nutrition Recommendations in the ScenoProt trial

Table S6 Participants' perceptions of their protein intake versus actual protein intake in relation to the Nordic Nutrition Recommendations in the ScenoProt trial

Table S7 Frequency of total counts of incidents when the category of the participant's perceived intake and the category of actual intake were the same after summing up the counts of the four macronutrients (carbohydrates, fibers, fats, and proteins) in the ScenoProt trial

Table S1 Carbohydrate and fiber sources named by participants and ingredient groups where carbohydrate sources were included in the ScenoProt trial

**Ingredient group**	**Included carbohydrate sources named by participants**	**Included fiber sources named by participants**
Vegetables (root vegetables and tubers, leaf vegetables, fruit vegetables, other vegetables)	Vegetables, root vegetables	Vegetables, root vegetables, salads
Nuts and seeds	–	Nuts, seeds, flaxseed groat, chia seed
Legumes	–	Legumes
Potatoes	Potato	–
Fruits and berries	Fruits, berries, apples, carrots	Fruits, berries, apple, bilberry
Cereals	Rice, pasta, whole-grain pasta, porridge, oatmeal, bran porridge, bread, rye bread, oat bread, grain, cereals, cereal products, breakfast cereals, muesli, couscous, oats, whole grains, bun, pastry	Cereals, porridge, oatmeal, spelt porridge, bran porridge, no-cook porridge, rice, pasta, whole-grain pasta, bread, whole-grain bread, rye bread, oat bread, brown bread, cereal products, whole-grain cereal products, whole-grain, bran, oat bran, rye bran, groats, oats, muesli, rye flakes
Fat (oil, margarine and vegetable fat spreads, butter, fat blends, and other fats, e.g., dressings, tallow, lard)	–	–
Fish and seafood	–	–
Meat (includes sausages and meat products)	–	–
Egg	–	–
Milk and dairy products	Quark	–
Plant-based dairy substitutes	–	–
Sugar, confectionery, and chocolate	Sugar, honey, chocolate, candied products, candy, confectionery, sweet treats	–
Other products	–	–
Beverages	–	–
Alcoholic beverages	–	–
	**Carbohydrate sources named by participants but not eligible for the analysis**	
	All food	

Table S2 Fat and protein sources named by participants and ingredient groups where the fat sources were included in the ScenoProt trial

Ingredient group	Included fat sources named by participants	Included protein sources named by participants
Vegetables (root vegetables and tubers, leaf vegetables, fruit vegetables, other vegetables)	–	Vegetables, quorn (Quorn's mycoprotein)
Nuts and seeds	Nuts, seeds	Nuts, seeds
Legumes	–	Beans, legumes, lentils, peas, chickpeas, plant protein products, Härkis (a registered trademark for faba bean product), soy, soy groat, tofu
Potatoes	–	–
Fruits and berries	Avocado	–
Cereals	Pastry	Grain, cereals, bread, pasta
Fat (oil, margarine and vegetable fat spreads, butter, fat blends, and other fats, e.g., dressings, tallow, lard)	Oil, cooking oil, vegetable oils, rapeseed oil, olive oil, hemp oil, coconut oil, sesame oil, butter, fat blends, margarine, light margarine, vegetable margarine, spread, light spread, dressing, fat used in cooking	–
Fish and seafood	Fish	Fish
Meat (includes sausages and meat products)	Meat, meat fat, meat products	Meat, red meat, meat products, sausage, chicken, broiler, animal protein
Egg	Egg	Egg
Milk and dairy products	Dairy products, cheese, fatty cheese, sour milk, milk fat	Dairy products, milk, skimmed milk, sour milk, yoghurt, cultured milk, quark, cheese, cottage cheese, whey, milk protein
Plant-based dairy substitutes	–	–
Sugar, confectionery, and chocolate	–	–
Other products	Fish oil capsules, fish oil	Supplements, recovery drink
Beverages	–	–
Alcoholic beverages	–	–
	**Fat sources named by participants but not eligible for the analysis**	
	Hidden fat	
	Goodies	
	Food	
	Fat in warm foods	
	Ready-made foods	

Table S3 Participants' perceptions of their carbohydrate intake versus actual carbohydrate intake in relation to the Nordic Nutrition Recommendations[a] in the ScenoProt trial (*n* = 102)

	Perceived intake		Actual intake	
	n (%)	95% CI, %	*n* (%)	95% CI, %
Below the recommendation (≤44.4 E%[b])	8 (8)	4.0–14.7	71 (70)	60.1–77.7
According to the recommendation (44.5–60.4 E%[b])	44 (43)	34.0–52.8	31 (30)	22.3–39.9
Above the recommendation (≥60.5 E%[b])	41 (40)	31.2–49.9	0 (0)	0.0–3.6
Answered: 'I do not know/cannot answer'	9 (9)	4.7–15.9	N/A	N/A

E%, a proportion from the total amount of energy; N/A, not applicable.

[a]Recommended carbohydrate intake range: 45–60 E% for adults.

[b]Alcohol excluded from energy.

Table S4 Participants' perceptions of their fiber intake versus actual fiber intake in relation to the Nordic Nutrition Recommendations[a] in the ScenoProt trial (*n* = 102)

	Perceived intake		Actual intake	
	n (%)	95% CI, %	*n* (%)	95% CI, %
Below the recommendation (≤24.4 g/d)	15 (15)	9.1–22.9	37 (36)	27.6–46.0
Below the recommendation (≤2.4 g/MJ[b])			19 (19)	12.3–27.3
According to the recommendation (≥24.5 g/d)	76 (75)[c]	65.3–82.0	65 (64)	54.1–72.4
According to the recommendation (≥2.5 g/MJ[b])			83 (81)	72.7–87.7
Answered: 'I do not know/cannot answer'	11 (11)	6.1–18.3	N/A	N/A

N/A, not applicable.

[a]Recommended fiber intake minimum 25–35 g/d for adults corresponding to approximately 3 g/MJ.

[b]Alcohol excluded from energy.

[c]Includes participants who answered either 'My intake is according to the recommendations' (*n* = 55; 54%) or 'My intake is above the recommendations' (*n* = 21; 21%).

Table S5 Participants' perceptions of their fat intake versus actual fat intake in relation to the Nordic Nutrition Recommendations[a] in the ScenoProt trial (n = 102)

	Perceived intake		Actual intake	
	n (%)	95% CI, %	*n* (%)	95% CI, %
Below the recommendation (≤24.4 E%[b])	4 (4)	1.5–9.7	1 (1)	0.2–5.4
According to the recommendation (24.5–40.4 E%[b])	40 (39)	30.3–48.9	64 (63)	53.1–71.5
Above the recommendation (≥40.5 E%[b])	51 (50)	40.5–59.5	37 (36)	27.6–46.0
Answered: 'I do not know/cannot answer'	7 (7)	3.4–13.5	N/A	N/A

E%, a proportion from the total amount of energy; N/A, not applicable.

[a]Recommended fat intake range: 25–40 E% for adults.

[b]Alcohol excluded from energy.

Table S6 Participants' perceptions of their protein intake versus actual protein intake in relation to the Nordic Nutrition Recommendations[a] in the ScenoProt trial (n = 86[b])

	Perceived intake		Actual intake	
	n (%)	95% CI, %	*n* (%)	95% CI, %
Below the recommendation (≤9.4 E%[c])	9 (10)	5.6–18.7	0 (0)	0.0–4.3
According to the recommendation (9.5–20.4 E%[c])	41 (48)	37.5–58.1	60 (70)	59.4–78.5
Above the recommendation (≥20.5 E%[c])	31 (36)	26.7–46.6	26 (30)	21.5–40.6
Answered: 'I do not know/cannot answer'	5 (6)	2.5–12.9	N/A	N/A

E%, a proportion from the total amount of energy; N/A, not applicable.

[a]Recommended protein intake range: 10–20 E% for adults aged 18–64 years.

[b]Includes participants aged 18–64 years.

[c]Alcohol excluded from energy.

Table S7 Frequency of total counts of incidents when the category of the participant's perceived intake and the category of actual intake were the same after summing up the counts of the four macronutrients (carbohydrates, fibers, fats, and proteins) in the ScenoProt trial (*n* = 102)

Total count	*n* (%)
0	15 (15)
1	28 (27)
2	38 (37)
3	15 (15)
4	6 (6)
